# Stochastic deposition of amino acids into microcavities via microparticles

**DOI:** 10.1038/s41598-019-52994-w

**Published:** 2019-11-11

**Authors:** Roman Popov, Girish Karadka Shankara, Clemens von Bojnicic-Kninski, Pramit Barua, Daniela Mattes, Frank Breitling, Alexander Nesterov-Mueller

**Affiliations:** Institute of Microstructure Technology, Karlsruhe Institute of Technology (KIT), Hermann-von-Helmholtz-Platz 1, 76344 Eggenstein-Leopoldshafen, Germany

**Keywords:** Chemical engineering, Other nanotechnology

## Abstract

All known methods for solid-phase synthesis of molecular arrays exploit positioning techniques to deposit monomers on a substrate preferably high densely. In this paper, stochastic patterning of molecule spots (250 000 spots monomers/cm^2^) via random allocation of the microbeads on a microstructured glass is presented. The size and shape of the microbeads and the microcavities are selected in such a way so that only one microbead can fit into the respective microcavity. Each microbead can be loaded with a certain type of molecule e.g. amino acids and is brought in the microcavities stochastically. Applying solvent vapor and heating the substrate, the molecules are released from the microbeads and coupled to the functionalized substrate. To differentiate between the microbeads carrying different molecules, quantum dot labels are preliminary introduced into the microbeads. Fluorescence imaging and subsequent data analysis enable decoding of the molecule deposition patterns. After the coupling step is completed, the microbeads are mechanically removed from the microwells. The composition of the monomer microbeads, their deposition and the conditions of the monomer extraction are studied. The stochastic monomer patterning may be used to design novel molecular arrays.

## Introduction

All techniques for solid-phase synthesis of molecular arrays are limited in terms of number of spots and require advanced positioning devices which tend to be more expensive the higher the spot densities are to be achieved. The reason for this is that generation of custom monomer sequences in individual spots requires prior information on where specific monomers have to be delivered for coupling to the substrate. Such a patterning of monomers can be achieved, for instance, by the lithographic masks^1,2^, digital micromirror devices^3^, laser scanning technique^4,5^, electrical fields of a computer chip^6^ or material printers^7^.

The only exception from these molecular arrays is oligonucleotide arrays, which can be generated by patterning of oligonucleotides stochastically. An example of such arrays is the bead arrays of Illumina^8,9^. First, oligonucleotides were synthesized on microbeads. Then, the microbeads loaded with molecules were stochastically deposited on a microstructured surface in array format for decoding^10^. These three principles, namely, stochastic deposition without predefined addressing of molecules, enrichment of these molecules in spots and the optical detection of monomers have determined the triumphal march of the oligonucleotide technique in the last decade, especially in the case of next-generation sequencers.

In this paper, we describe particles loaded with molecules such as amino acids that may enable the realization of these three principles for *in situ* synthesis of high-density molecular arrays. Therefore, we focus on assembly of microparticles on the micro-structured surfaces (stochastic deposition), on the extraction of amino acids from these particles (amplification in spots) and on deriving the allocation of the monomers using quantum dot (QD) labels of the particles (optical detection). The polymer solid particles with embedded amino acids were reported for synthesis of peptide arrays via laser printer^11^. As these particles were produced via milling processes, they possessed a relatively broad size distribution and unregular form. In contrast, the proposed stochastic deposition is based on the use of the monodisperse microparticles.

The assembly of microparticles on micro and nanostructures was intensively studied for biochip devices and sensors^12–16^. In these applications, microarrays using color-encoded beads were sucessfuly demostrated^17,18^. According to our knowledge, we first demonstrate the assembly of particles that can deliver monomers for solid phase synthesis in microstructures in high-density array format.

## Principle: Particles As Carriers Of Amino Acids

**Figure 1:**
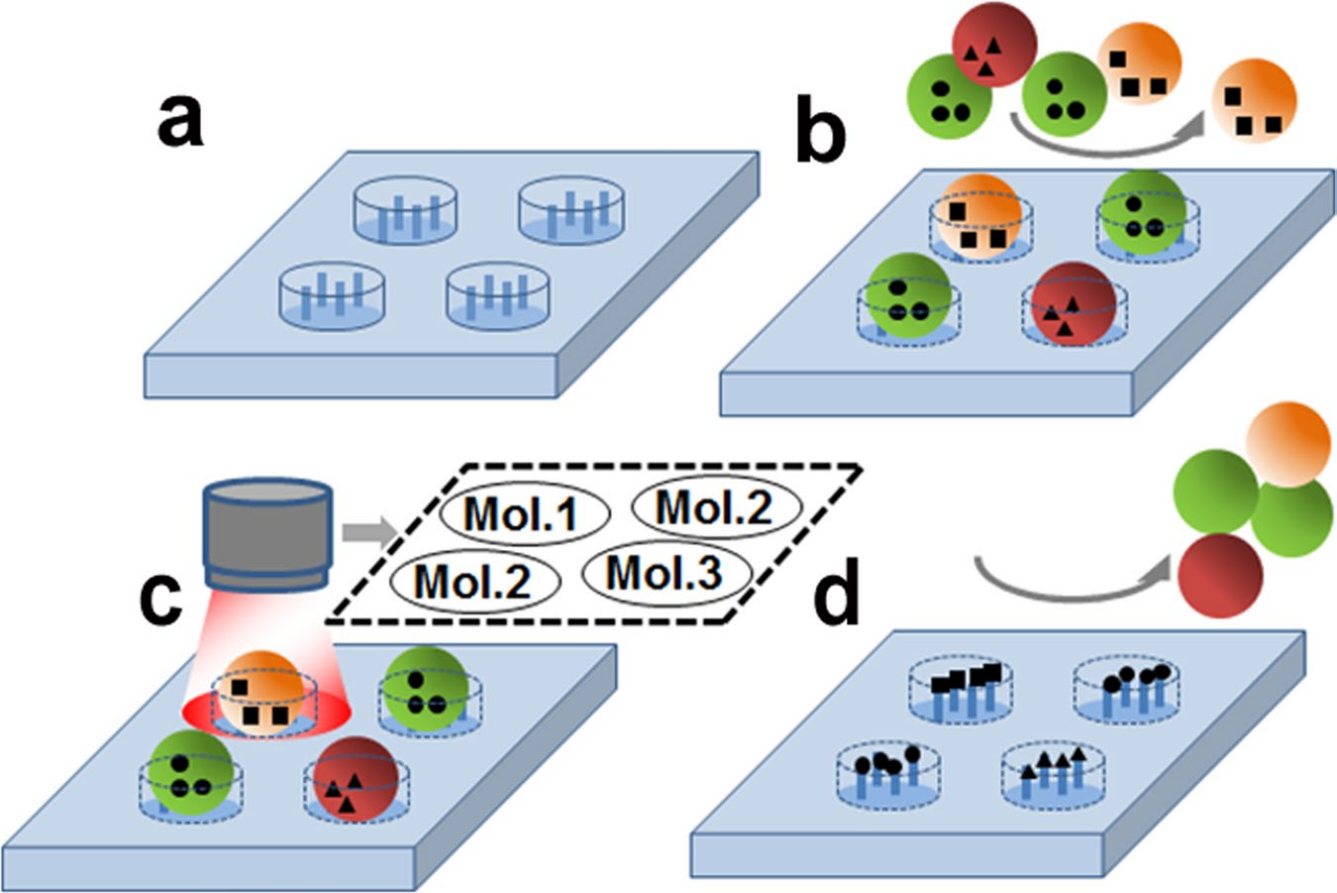
Schematic illustration of stochastic patterning of molecules. (**a**) Substrate with microcavities functionalized with free amino groups; (**b**) stochastic deposition of a mixture of microbeads loaded with different molecules (Mol.); (**c**) decoding of molecule patterns; (**d**) extraction and solid phase coupling of the molecules and removal of the microbeads.

Figure 1 illustrates the functionalities of the particles proposed in this study^19^. The patterning of the particles occurs on a microstructured glass surface. The microcavities have a cylindrical form. The glass surface is functionalized with free amino groups which can react with the activated carboxyl groups of molecules, for example amino acids, forming peptide bonds.

The size and shape of the microbeads and the microwells are selected in such a way so that only one microbead can fit into the respective microwell. Such a geometric constraint does not allow more than one type of molecules to be present in each microwell during the coupling step. As a result, the array spots are expected to contain predominantly individual kinds of the molecule after their stochastic patterning is completed.

The generation of the molecular patterns takes place in a single step by mechanically applying a mixture of different microbeads into the microwells of the microstructured substrate. Since the deposition of the microbeads is performed in a stochastic process, it is not known in advance which type of microbeads will be located in each microwell. To differentiate between the microbeads carrying different monomers, specific fluorescent labels are preliminary introduced into the microbeads. Fluorescence imaging and subsequent data analysis enable decoding of the monomer deposition patterns.

Under certain conditions, the molecules are released from the microbeads to enable their diffusion to the functional layer and further coupling to the terminal free amino groups. After the coupling step is completed, the excess of molecules and the process by-products are washed away, whereas the microbeads are mechanically removed from the microwells.

## Results and Discussion

### Particle design

**Figure 2:**
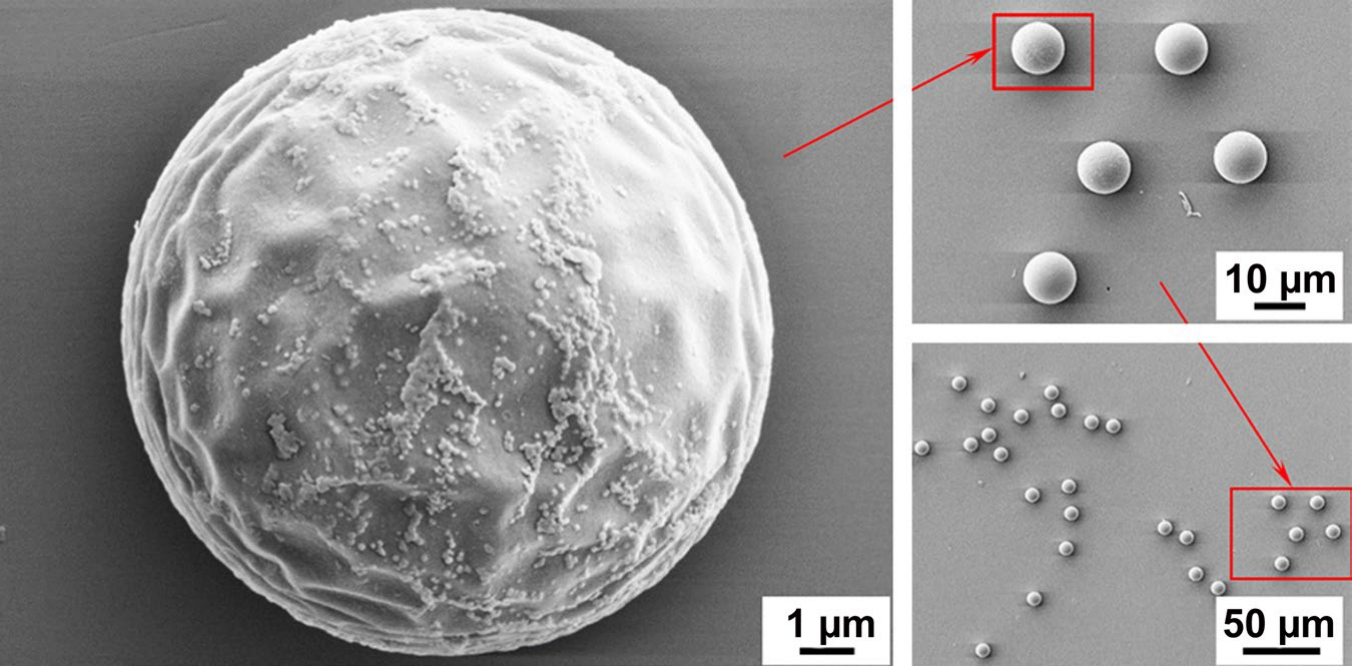
Scanning Electron Microscope (SEM) images of cross-linked PMMA microspheres.

The microbeads developed within the framework of the present work are based on the solid-carrier architecture. Special polymer-based microspheres were used as microcarriers of the amino acid derivatives and QDs. The cross-linked poly(methyl methacrylate) (PMMA) microspheres were selected as solid carriers of the amino acid derivatives and QDs. They were manufactured by emulsification polymerization and cross-linking with 3% divinylbenzene according to the internal protocols of the company. The microspheres had a mean diameter of 10 μm with an extremely narrow size distribution. The coefficient of variation (CV) was declared to be less than 5% (Fig. 2).

The commercially available amino acid derivative Fluorenylmethoxycarbonyl-glycine pentafluorophenyl ester (Fmoc-Gly-OPfp), were used as monomer. Hydrophobic ZnCdSeS alloyed QDs were used as fluorescent markers for labeling the microbeads. The core of the QDs was coated with hydrophobic organic molecules, which get them readily suspended in non-polar organic solvents. The quantum dots with the emission maximum at 470 nm, 490 nm, 500 nm, 520 nm, 530 nm, 550 nm, 560 nm, 570 nm, 580 nm, 590 nm, and 610 nm were tested. The declared accuracy of the maximum emission wavelength was +/−5 nm. The full width at half maximum (FWHM) of the QD emission spectra was 35 nm.

The process of microbead manufacturing is carried out in two stages. In the first stage, the solid carriers were labeled with individual QD combinations. For this purpose, the solutions of QDs in chloroform (2.5 mg/mL) were sequentially added in equal volumes to the dispersion with PMMA microspheres. To derive the optimum content of the QD labels per 1 g of solid carriers, the total volume of the QD solutions varied in the range between 50 μL and 150 μL. The mixture of the microspheres and QDs in dichloromethane (DCM) was stirred in the closed vial for another 30 min to reach the homogeneous state. While stirring, the hydrophobic QDs were distributed in the continuous phase with the relative permittivity close to that of pure DCM (εr = 8.9). On the next step, the vial was opened to initiate evaporation of DCM (TB = 40 °C). During stirring, 30 mL of acetone (εr = 20.7) were added dropwise to the dispersion from a burette. The feeding rate of acetone was adjusted in such a way so that the volume of the dispersion was kept constant and equal to approximately 11 mL. The intention was to change gradually the composition of the continuous phase since the evaporation rate of acetone (T_B_ = 56 °C) is smaller than the evaporation rate of DCM. As the content of acetone increased, the polarity of the continuous phase increased as well. The hypothesis was that the gradual increase of the polarity of the continuous phase induced precipitation of the hydrophobic QDs onto the microspheres that remain swollen in acetone. In the next step, 30 mL of ethanol (ε_r_ = 24.5) were added dropwise to the dispersion from the burette. The feeding rate was adjusted so that the volume of the dispersion remained constant. Due to the fact the evaporation rate of ethanol (T_B_ = 78 °C) is smaller than that of acetone and DCM, its content in the continuous phase had to increase over time. It induced a gradual contraction of the cross-linked PMMA microspheres, resulting in immobilization of the QDs within the outer polymer layer. As the stirring process was terminated, the microspheres sedimented from the continuous phase. The supernatant was carefully removed with a pipette, followed by washing of the microspheres with ethanol and acetone to eliminate excessive QDs. Finally, the microspheres were dried to remove the solvent residues.

On the second stage, the amino acid derivatives were introduced into the polymer matrix of the labeled solid carriers. A sample of amino acid derivatives was initially dissolved in 10 mL of DCM. The mass of the amino acid derivatives was varied in the range between 10 mg and 50 mg with the aim to define the optimum monomer content in microbeads. The amino acid solution was added to 1 g of QD labeled microspheres. The resulting dispersion was gently stirred until a paste-like medium was obtained due to gradual evaporation of the solvent from the open vessel. The paste dried up slowly over 2 hours. The resulting dry mass of microbeads was milled in a falcon tube with several metal spheres (Ø = 5 mm) on a vortex shaker.

Three assumptions were made in favor of the method’s viability. First, the microbeads swelled in DCM within a relatively short time. Second, the QDs embedded into the microspheres in a previous stage remained inside the cross-linked polymer without being extracted. Third, the amino acid derivatives gradually precipitated from the saturated solution into the swelled microbeads, while the solvent underwent evaporation.

Despite the fact the microbeads were subjected to the mechanical impact and rubbing against the surface of the substrate, they kept the embedded amino acid residues and did not produce any contaminations over the surface. In contrast to this, the sample with 8% (w/w) of Fmoc-Gly-OPfp and 6% (w/w) of *N*,*N*- Diphenylformamide (DPF) was characterized by numerous impurities of the amino acid derivatives in form of scratch-like lines and non-circular spots with the size of several micrometers.

As soon as the total mass fraction of the embedded components exceeds 8% (w/w), the microbeads tend to contaminate the surface with the residues of amino acid derivatives. We assume that these impurities originate from the excessive amino acids and DPF crystallized on the surface of the microbeads during their manufacturing due to the fact the outer thin layer of the cross-linked PMMA matrix is already occupied by these substances.

Similar experiments were performed for the remaining 19 types of proteinogenic amino acids, whereas only the content of the monomers was varied in mass fraction of 2% (w/w), 3% (w/w), 4% (w/w), 6% (w/w) and 8% (w/w). The mass fraction of the monomers in these microbeads are listed in Supplementary Table S1. To be mechanically stable, the cross-linked PMMA solid carriers could accommodate the amino acids in mass fractions 3%(w/w) and below. Thus, the mechanical stability appeared to be depended on the availability of functional side change groups.The results of this experiment demonstrate the dilemma of microbead deposition: A low mechanical impact leads to insufficient filling rates or high contamination of the top surface with excessive microbeads, whereas great mechanical forces damage the microbeads making them not applicable for the stochastic peptide microarray manufacturing.

### Particle deposition and removal from the microstructures

The full-size microstructured substrates were made of fused silica using photolithography and Reactive-ion etching (RIE) and had dimensions of a standard microscopic slide. The microstructures with the diameter of 11 μm, 12 μm and 13 μm and the depth of 9 μm and 10 μm were compared in terms of their filling rates after depositing the microspheres with the diameter of 10 μm.

**Figure 3:**
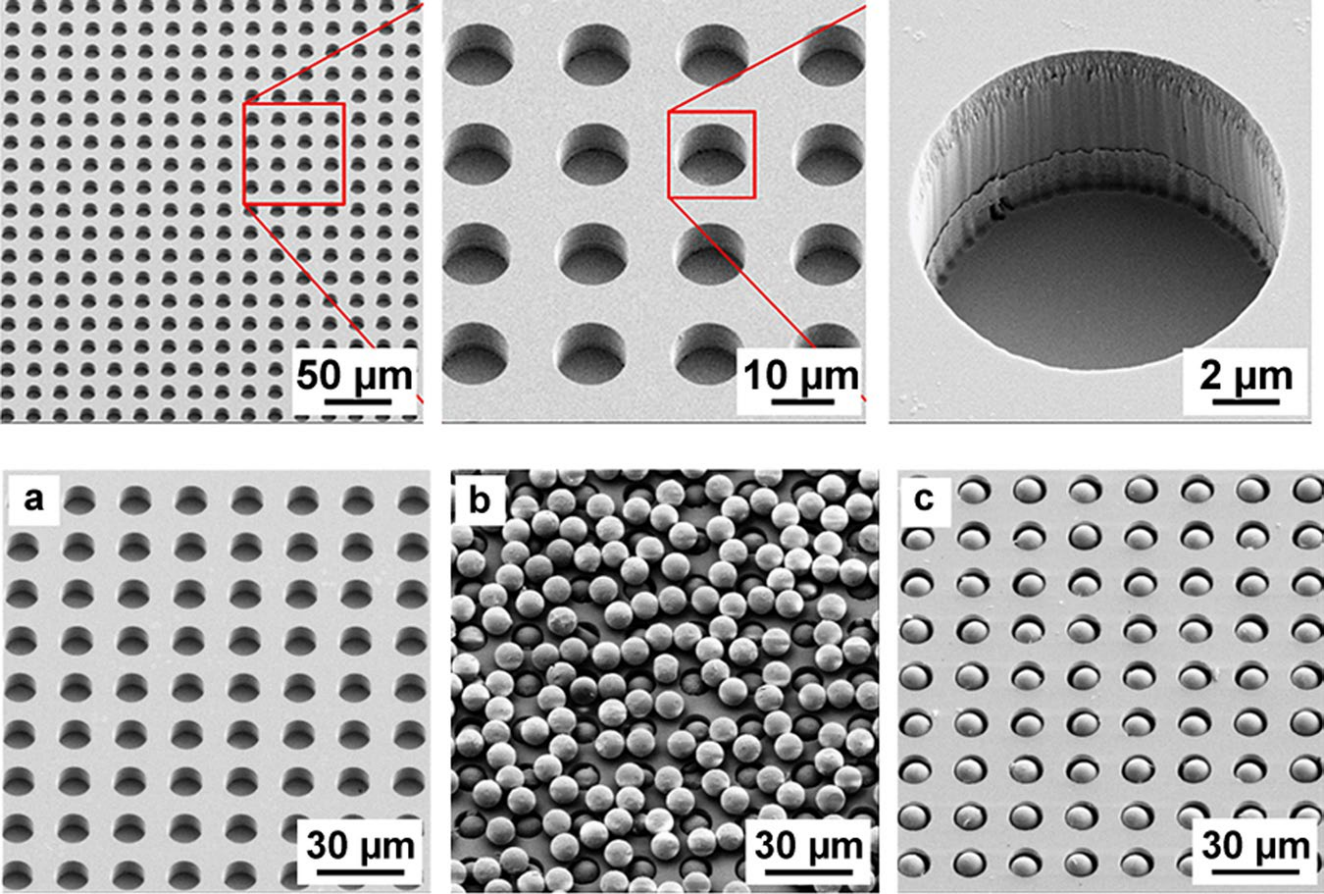
(top) SEM images of the microstructured substrate. (bottom) SEM images of a microstructured substrate demonstrating the principle of assembly of the microbeads: (**a**) initial empty microstructures; (**b**) microstructures after spreading the microbeads with a soft tissue; (**c**) microstructures after applying a tangential flow of compressed air.

The deposition of the microbeads was performed in two steps. First, the dry mixture of the microbeads was spread over the surface of the microstructured substrate (Fig. 3a) with a soft lint-free tissue. Although the microstructures were completely filled with the microbeads, the top surface of the substrate still contained a monolayer of excessive microbeads (Fig. 3b). This excess was removed in the second step with the flow of compressed air applied tangentially to the surface of the substrate. The air flow totally removed the excessive microbeads from the top surface, whereas the microwells remained filled (Fig. 3c).

The filling rate of various microstructures strongly depended on the microcavity geometry. It was derived from the analysis of the acquired optical images and is shown in Supplementary Table S2. A filling rate of 98.4% was observed at optimum parameters.

Another factor, which had to be taken into account when deciding on the optimal parameters of the microstructures, was the microbead removal rate. Removal of the microbeads from the microwells was performed in several steps by placing the substrate in a liquid medium and exposing it to acoustic waves in an ultrasonic bath. In the first step, the substrate was placed into the falcon tube filled with a mixture of 5% (v/v) *N*-methylethylamine (MEA) and acetone and sonicated for 2 min. MEA was used as a passivation additive to avoid contamination of the microwells with a mixture of non-coupled amino acids derivatives. In the next step, the substrate was sonicated. The microbead removal rate was analyzed by optical microscopy and scanning with the fluorescence scanner using neutral-density filters.

The close geometric matching of the non-soluble polymer microspheres to the microwells resulted in extremely low rates of their removal. If the size of the microsphere matched the diameter of the microwell, it was almost impossible to remove this microsphere without deforming or destroying it. Although the cross-linked PMMA microspheres had a mean size of 10 μm, the coefficient of variation of their diameter was declared to be 5%. It implies that roughly 1% of the microspheres had a diameter ranging from 10.9 μm to 11.0 μm (assuming normal size distribution) that could potentially block thousands of microwells with the diameter of 11 μm in each step of microbead deposition.

Taking into account the results obtained in a series of experiments, the decision was made to continue the experimental work with the full-size microstructured substrates (75 mm × 25 mm × 1 mm) having the pitch size of 20 μm, the microwell diameter of 12 μm and the depth of 9 μm (filling rate 90.2%).

Assembly of the microbeads in the microwells of the microstructured substrate is one of the fundamental principles of the stochastic peptide microarray manufacturing. Microbead deposition into the microwells of the substrate was prone to various errors. The most frequent cases of deposition errors are illustrated in Supplementary Fig. S1. Unfilled microwells lead to the termination of molecule array synthesis due to the absence of monomers in a certain step of peptide chain elongation. It results in a reduction of the number of fully-synthesized oligomers. In case of the microbeads left on the top surface of the substrate, the major adverse effect is the contamination of the neighboring microwells by arbitrary amino acid derivatives extracted from the residual microbeads. It locally affects the quality of the synthesized oligomers and can lead to false positive signals in biological applications. The microbead, “hanging” on the side wall of the microwell and not touching its bottom, is only partially introduced into the microwell. The monomer source point is shifted outside of the area where the oligomers are supposed to be synthesized namely at the bottom of the microwell. Eventually, it may result in false negative signals when implementing a molecular microarray in bioassays.

### Decoding of amino acid positions via QD-labelled microbeads

The size of the microbeads imposes a restriction on the spatial resolution of the digital images acquired by the optical system. To enable an accurate calculation of the photometric data for each microbead, the image resolution has to be at least 2 μm/pixel. Image acquisition was performed with a confocal fluorescence scanner InnoScan 1100 AL (Innopsys). The scanner was equipped with three excitation laser sources (488 nm (blue channel), 532 nm (green channel), and 635 nm (red channel)). The scanner had the optical resolution of 0.5 μm/pixel and used a real-time autofocus system. A grid with predefined parameters corresponding to the layout of the microstructured substrate was applied to the image and automatically adjusted to match the microwell pattern. Each spot of the substrate underwent automatic segmentation and was analyzed in each fluorescence channel to derive the photometric feature and background values. The photometric data calculated for all the spots were saved in a file.

**Figure 4:**
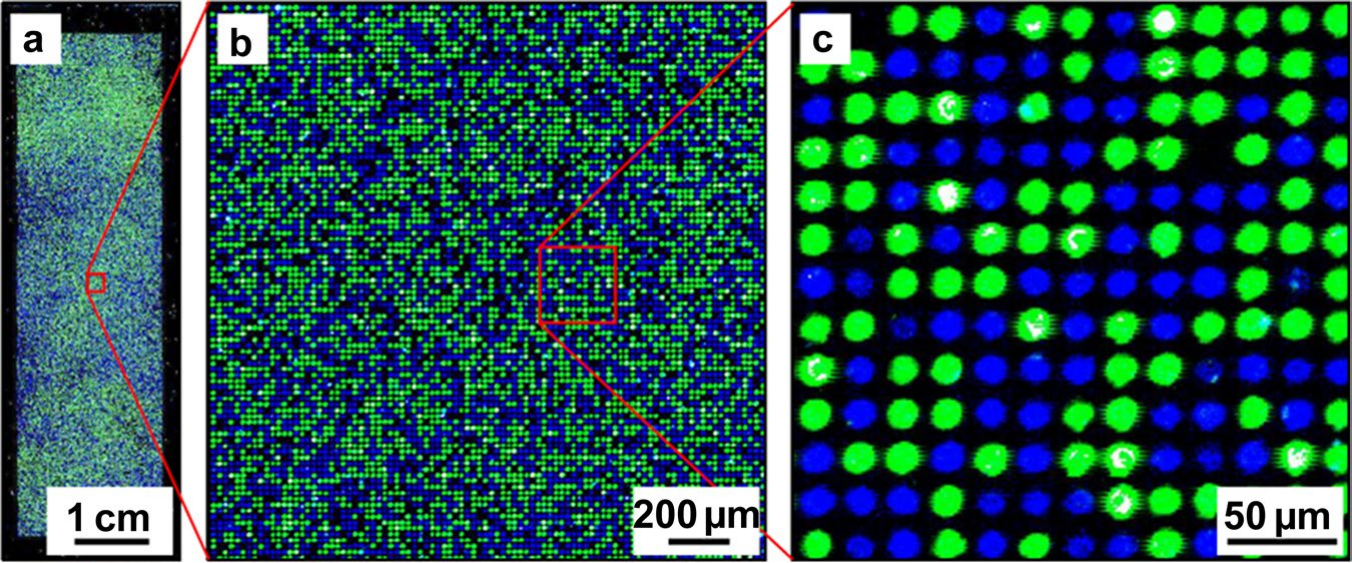
Deposition pattern of microbeads labeled by 500 nm QDs (shown in blue) and 580 nm QDs (shown in green) in the microwells of the microstructured substrate. The microbead deposition was performed using a soft lint-free tissue and a compressed air flow: (**a**) full-size image; (**b**) (**c**) image fragments.

In the next iteration, a set of microbeads labeled with individual types of QDs with emission maximum wavelengths ranging between 490 nm and 610 nm were manufactured and scanned in the fluorescence channels defined in the previous step. The mean and the standard deviation of the median feature values were estimated for each type of QD labeled microbeads in every fluorescence channel based on 200 sample spots. The optimal set of QD labels for the respective fluorescence channels was defined based on detection specificity. While the optical distinguishing between particles labeled with different single QDs was easily (Fig. 4a–c), the optical detection of multiple labeled particles required special combinations which can be derived from the Supplementary Table S3.

By using four types of basic QDs, in total 14 types of QD labeled microbeads were manufactured (Table 1).

**Table 1:** Possible combinations of basic QDs for microbead labeling.

QDs, nm	QD combinations													
	1	2	3	4	5	6	7	8	9	10	11	12	13	14
500	x	—	—	—	x	x	x	—	—	—	x	x	—	x
550	—	x	—	—	x	—	—	x	x	—	x	x	x	x
580	—	—	x	—	—	x	—	x	—	x	x	—	x	x
610	—	—	—	x	—	—	x	—	x	x	—	x	x	x

**Figure 5:**
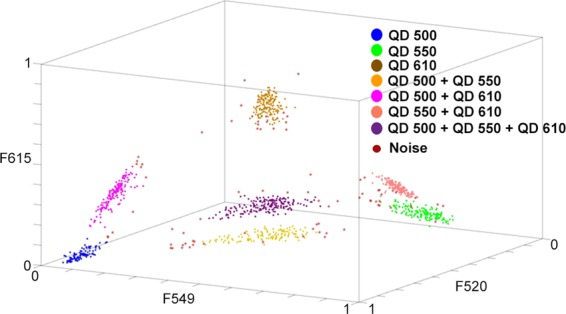
DBSCAN clustering of seven types of QD labeled microbeads: allocation of data points according to the microbead signals normalized for three fluorescence channels.

The method of unsupervised learning was used the task of microbead classification: density-based spatial clustering of applications with noise^20^. It employs the principle of density-based clustering: The objects closely packed in a given space are grouped together into a cluster, whereas the objects which are located in low-density regions are considered to be outliers. Figure 5 demonstrates DBSCAN clustering results on an expanded set of QD labeled microbeads. Three types of microbeads labeled with individual basic QDs were supplemented by four types of microbeads labeled with QD combinations. As can be seen from Fig. 5, four additional clusters fit in well between the clusters of the mono-labeled microbeads. Moreover, each cluster of the multi-labeled microbeads is located between the clusters of the microbeads labeled with the corresponding basic QDs. Since each data point represents the microbead signal normalized for three fluorescence channels, the clustering problem becomes two-dimensional. To confirm this, one can see that all seven clusters are located on the major diagonal plane (F520 + F549 + F615 = 1).

After scanning all 14 microbeads in the corresponding fluorescence channels, the maximum number of microbeads resolved by DBSCAN was identified to be 11. The experiment was planned in such a way so that the types of the microbeads were known in advance. It enabled an estimation of the error rates of identifying the microbeads as outliers, as well as the error rates of identifying the microbeads as of another type (see Supplementary Table S4). As can be seen in Table S4, the error rates of identifying the microbeads as outliers were significant and ranged between 0.5% and 6.7%. At the same time, the error rates of identifying the microbeads as of another type were relatively low (0.2%). The error rates could considerably decrease by improving the homogeneity of the QD labeled microbeads, by implementing an additional fluorescence filter, or by decreasing the number of microbead types.

### Extraction and coupling of amino acids

The microstructured slide were functionalized with a thin copolymer layer of 10% poly(ethylene glycol) methacrylate (PEGMA) and 90% methyl methacrylate (MMA), respectively. The thickness of the functional layer ranged between 10 nm and 15 nm, whereas the surface density of the amino groups ranged between 1.5 nmol/cm^2^ and 2.5 nmol/cm^2^ ^21^.

**Figure 6:**
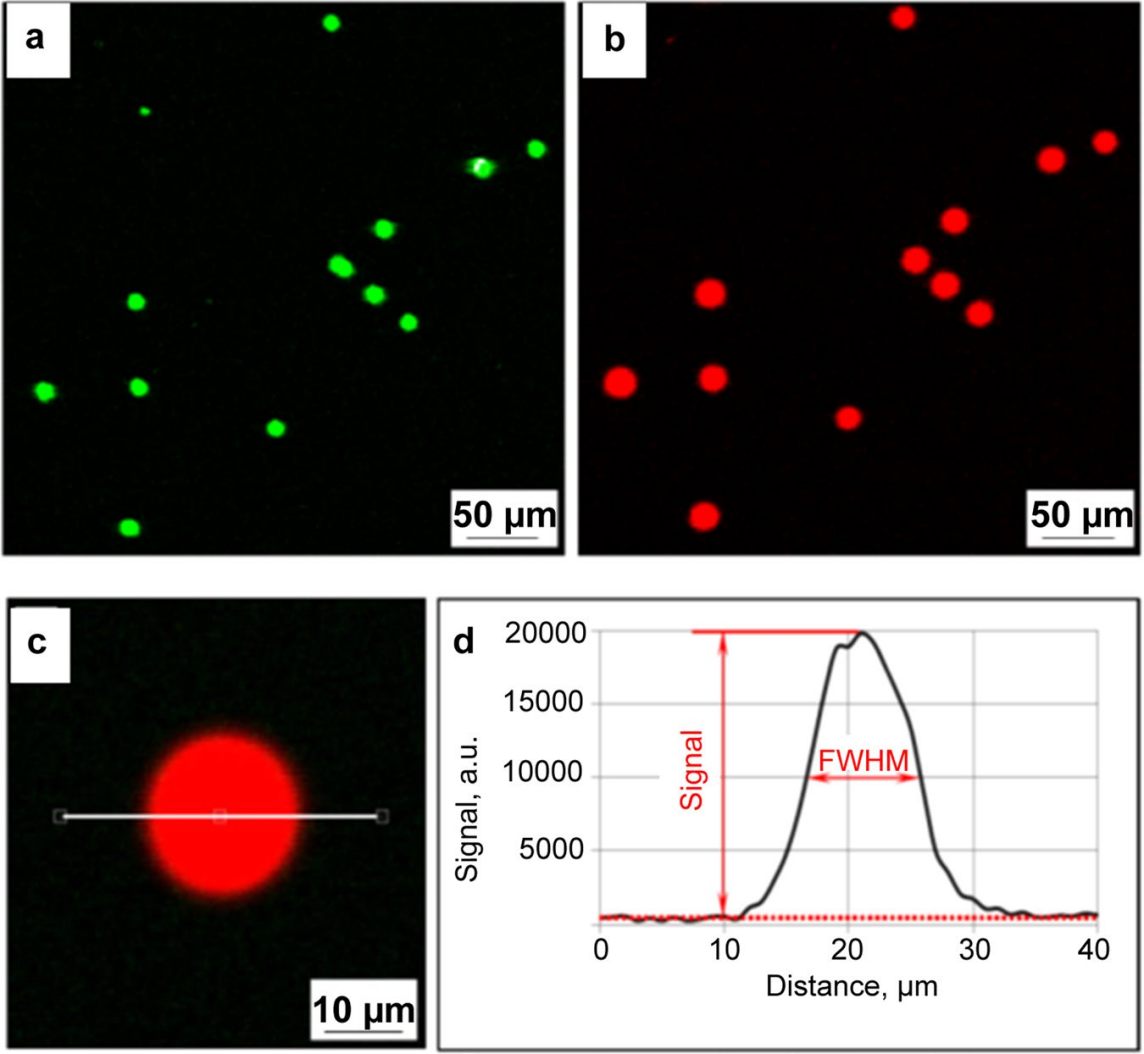
(top) Coupling results of the microbeads labeled with 580 nm QDs and loaded with Fmoc-Gly-OPfp; (**a**) random pattern of the QD labeled microbeads deposited onto a flat functionalized substrate. The image was acquired in the fluorescence channel 580/14 nm (green channel); (**b**) fluorescently labeled amino acids extracted from the microbeads and coupled to the functional surface. The image was acquired in the fluorescence channel 677/45 nm (red channel); (bottom) Cross section profile of an amino acid spot; (**c**) fluorescence image of an amino acid spot with a cross-section line; (**d**) a cross-section profile of the amino acid spot.

The function of the microbeads as carriers of the amino acid derivatives is demonstrated in Fig. 6. The microbeads were labeled with 580 nm QDs and loaded with Fmoc-glycine pentafluorophenyl ester (Fmoc-Gly-OPfp). After random deposition of the microbeads on the flat functionalized substrate, they were imaged in the fluorescence channel 580/14 corresponding to the emission maximum of the QD labels (Fig. 6a). Thereafter, the amino acid derivatives were extracted from the microbeads and coupled to the functional layer of the substrate. After microbead removal, surface acetylation and Fmoc-deprotection, the coupled amino acids were fluorescently stained with *DyLight* 650 NHS ester dye. It made it possible to visualize the coupled monomers in the fluorescence channel 677/45 corresponding to the emission of the fluorescent dye (Fig. 6b).

Each spot was characterized by two parameters that were derived from its cross-section profile measurement (Fig. 6c,d). In most cases, the spot profile could be approximated by a Gaussian function. The height of the background-corrected bell curve was treated as a spot signal and was considered proportional to the surface concentration of the coupled amino acids. The higher coupling yields were associated with stronger spot signals, provided that other factors remained the same. The coupling yield was estimated as the ratio between the spot signal and the reference signal associated with an excessive amount of monomers coupled from their solution in N,N-Dimethylformamide (DMF). Another characteristic value, the diameter of the amino acid spot, was assigned to the Full Width at Half Maximum (FWHM) of the background-normalized bell curve. The spot diameter defined the degree of amino acid diffusion during their extraction and coupling.

The extraction process took place in a saturated vapor of DCM. A special metal chamber was designed to facilitate the extraction process (Fig. S2). It was made of stainless steel and included two polytetrafluoroethylene (PTFE) tanks for the liquid-state organic solvent. After adding 30 mL of DCM to the tanks, the chamber was kept closed for 30 min to achieve the equilibrium state. Thereafter, the substrates were inserted into the chamber for a certain period of time. After taking the substrates out of the chamber, the process was repeated several times to enhance the extraction of the amino acid derivatives from the microbeads. The subsequent coupling step was performed for 60 min at 90 °C. For this purpose, the substrate was placed into a coupling chamber, which was filled thereafter with argon and put in the preheated oven. After removing the coupling chamber from the oven, it was slowly cooled down to ambient temperature.

The duration of the amino acid extraction in the solvent chamber was optimized for microbeads containing 5% (w/w) of Fmoc-Gly-OPfp. Amino acid extraction was performed five times for the periods of 0.5 min, 1.0 min and 2.0 min each with intermediate pauses of 1 min. Thereafter, the substrate was placed in the oven and stained as described above. After fluorescence scanning, the mean spot signals and spot diameters were estimated (see Supplementary Table S5).

The data shown in Table S5 contradicts to the hypotheses that the longer extraction times result in higher coupling yields and larger spot diameters. Indeed, the mean spot diameter increases as the extraction period becomes longer, whereas the spot intensity gradually abates. The longer the substrate remains in the saturated gas atmosphere, the larger amount of organic solvent condensates at the contact point of the microbead to the surface. It enhances the swelling of the microbead, the release of the amino acid derivatives, as well as their diffusion over the functional layer. Due to their spreading over the functional layer, the surface concentration of the amino acid derivatives decreases, resulting in a lower spot intensity. In fact, the integral signal over the spot area, associated with the total amount of the extracted and coupled monomers, increases as the process duration becomes longer. Therefore, shorter process durations are preferable if the diameter of the amino acid spots ranges between 15 μm and 30 μm. The spot size constraints are due to the fact that the monomers, extracted from the microbeads, should homogeneously spread over the bottom of the microwell and not diffuse to the neighboring microstructures. This requirement was met in case the amino acid extraction period was 1 min.

In order to enhance the coupling of the amino acid derivatives, it was suggested to perform the extraction process multiple times for 1 min each. Microbeads with 5% (w/w) of Fmoc-Gly-OPfp were deposited onto several flat functionalized substrates, which were independently placed into the extraction chamber 3, 5, 7, 10, and 15 times for 1 min each with their intermediate removal for 1 min. The resulting mean spot signals and diameters were measured and listed in Supplementary Table S6.

The initial hypothesis was that in each repetition, an additional amount of amino acids derivatives could be extracted from the microbeads, resulting in a higher coupling yield without considerable spreading of the monomers. The data shown in Table S6 indicates that the correlation between the spot signal and the number of process repetitions is more complex than the direct relationship between the spot diameter and the number of process cycles. By increasing the number of repetitions from 3 to 15, the spot diameter gradually increased from 15.3 μm to 30.1 μm, whereas the spot intensity first increased from 24.3∙10^3^ a.u. to 34.4∙10^3^ a.u. for 5 repetitions and then slowly decreased to 27.8∙10^3^ a.u. Starting from 5 repetitions, the spot signal decreased due to the fact the extracted amino acid derivatives diffused over a larger area defined by the spot diameter. In case of 3 repetitions, the amino acid extraction rate was not yet sufficient to result in a high surface density of the monomers. Hence, the optimum confinement of the amino acid derivatives within the microwells could be achieved by extracting the monomers 5 times for 1 min each.

**Figure 7:**
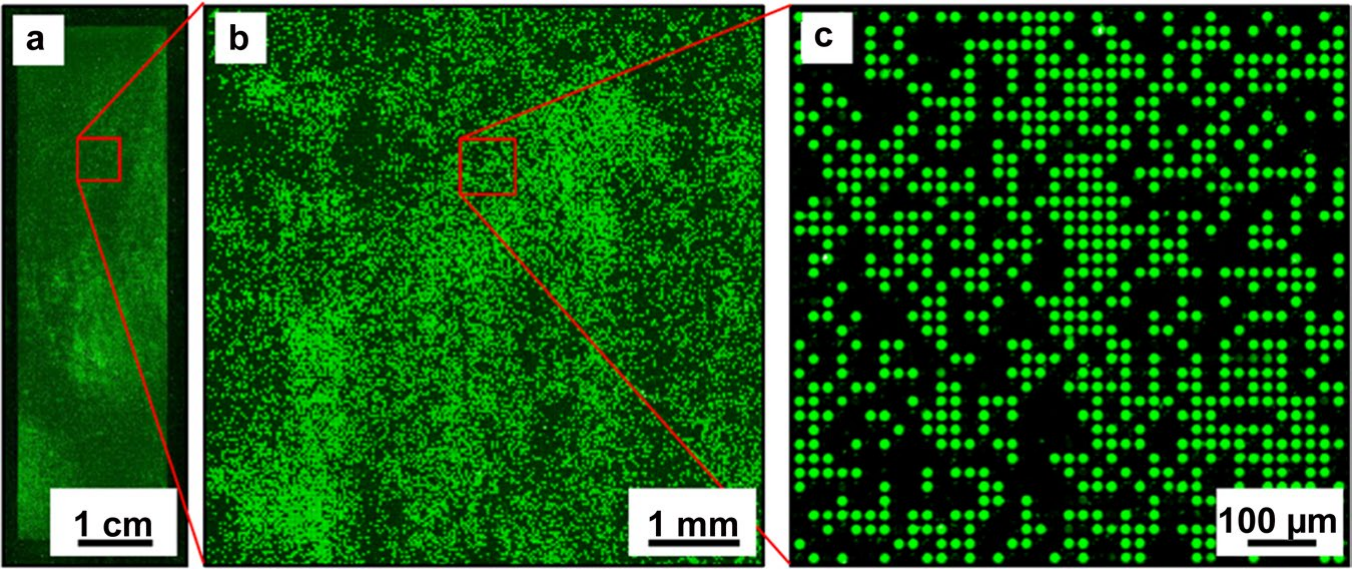
Fluorescence pattern generated by stochastic deposition of microbeads carrying Fmoc-Gly-OPfp and subsequent extraction and coupling of the monomers in the microwells of a microstructured substrate, followed by fluorescence staining with TAMRA: (**a**) full-size image; (**b**) (**c**) image fragments. The fluorescence signal is proportional to the surface concentration of the coupled amino acid derivatives. The filling rate was reduced on purpose to verify the events of amino acid diffusion into the neighboring empty microwells.

The optimum extraction parameters were tested on microbeads with 5% (w/w) of Fmoc-Gly-OPfp deposited into the microwells of the microstructured functionalized substrate. Extraction of the amino acid derivatives was performed in the chamber with saturated DCM atmosphere for 1 min periods repeated 5 times with intermediate removal of the substrate for 1 min. The coupling, fluorescence staining and imaging were performed according to the standard procedures. The resulting fluorescence pattern is depicted in Fig. 7. The filing rate of the microstructures was intentionally kept low to verify, whether the amino acid derivatives diffused to the neighboring microwells during extraction and coupling steps. The qualitative analysis of the fluorescence image indicates that the extracted amino acids were confined to the microwells, whereas their diffusion over the top surface was prevented. These results confirm that the parameters of the amino acid extraction optimized for the flat functionalized substrates were also suitable for the microstructured surfaces. The coupling efficiency after extraction was determined by comparing the fluorescent signals after staining free amino groups of glycine monomers coupled from particles and from solution on different parts of the same substrate. The coupling efficiency in the case of Glycine particles was 84% of the Gly coupling efficiency from the solution (see Supplementary Fig. S3).

In a similar way, the optimum extraction conditions were confirmed for all 20 types of microbeads carrying different proteinogenic amino acid derivatives. The microbead samples with the amino acid content defined in Table S1 were individually deposited into the microwells of the microstructured substrate. Amino acid extraction was performed in DCM chamber 5 times for 1 min each with intermediate removal of the substrate from the chamber for 1 min. The coupling, staining, and fluorescence imaging resulted in amino acid spots confined to the microwells of the microstructured substrate, which enabled us to conclude that the optimum extraction parameters were in general suitable for the stochastic patterning of proteinogenic amino acids.

## Methods

### Microbead manufacturing

1 g of cross-linked PMMA microspheres (Spheromers® CA10, Microbeads AS) was dispersed in 10 mL of DCM in a 20 mL vial. The vial with a cap was closed to prevent evaporation of DCM. The dispersion was gently stirred for 30 min to achieve swelling of the microspheres. Then, 100 μL of QD (PlasmaChem GmbH) solution in chloroform (2.5 mg/mL) was added and the dispersion was stirred in the closed vial for 30 min. Dispersion from the vial was replaced to a 25 mL beaker. 30 mL of acetone was added dropwise from a burette. The feeding rate was adjusted in such way that the liquid level remains constant (i.e. overcomes the evaporation rate of the solvents). Afterwards, 30 mL of ethanol was added dropwise from a burette and the feeding rate was adjusted in such way that the liquid level remains constant or slowly increases. After stirring, the microspheres sedimented onto the bottom. The supernatant was removed with a pipette. Then, 20 mL of ethanol was added and the dispersion was stirred for 2 min. After stirring, the microspheres sedimented. Again, the supernatant was removed with a pipette. This washing step with ethanol was repeated two times. The next washing step with acetone (20 mL of acetone, stirring time 2 min) was repeated two times.

To load the beads with a monomer, 4 mL solution of Fmoc-protected OPfp-activated amino acid (Merck KGaA, Bachem AG) in DCM was added to the beads. The mass concentration (%, m/v) of amino acid in the solution was adjusted for each type of monomer (see Supplementary Table S7). The dispersion was gently stirred until a paste-like medium was obtained. After stirring, the paste was dried out completely. The dry particle agglomerates were transferred to a falcon tube with 2 steel beads (Ø 5 mm) and milled using a vortex shaker until a fine powder was obtained. To store the powder in a freezer, it was placed into a falcon tube, filled with argon and sealed with Parafilm.

## Coupling Of Amino Acids

### Fmoc deprotection

The microstructured substrate was functionalized with free amino groups according to the protocol^13^ and before using washed in *N*,*N*-Dimethylformamide (DMF) for 5 min. Then, a solution of 20% (v/v) piperidine in DMF was applied for 30 min. Afterwards, the substrate was washed two times in DMF for 5 min each, two times in methanol for 3 min each and finally in DCM for 1 min.

### Microbead deposition and image acquisition

A mixture of microbeads was applied onto the substrate and spread over the surface with a lint-free tissue (Kimtech Science, Kimberly-Clark). The excessive microbeads were removed from the top surface using a compressed air flow. An intermediate control of the microstructures’ filling rate was performed using optical microscopy. The fluorescence scanning of the substrate was carried out in the fluorescence channels corresponding to the emission wavelengths of the QD labels. The images acquired with the fluorescence scanner were analyzed using Mapix software (Innopsys).

### Amino acid extraction and coupling

The substrate was placed into the extraction chamber with a pre-saturated DCM atmosphere five times for 1 min each with intermediate removal of the substrate from the chamber for 1 min. Thereafter, the substrate was placed into the coupling chamber filled with argon. Afterwards, the coupling chamber was placed in the preheated oven for 60 min at 90 °C. The chamber was removed from the oven and cooled down to room temperature.

### Microbead removal

The substrate with microbeads was washed sequentially in a solution of 5% (v/v) MEA in acetone for 2 min, in acetone for 3 min, and in DCM for 2 min, each time in an ultrasonic bath. After washing, the substrate was dried and the microbeads removal rate was controlled using optical microscopy.

### Capping free amino groups

After removal of microbeads, the substrate was washed in DMF for 5 min. Then, 10 mL solution of 10% (v/v) acetic anhydrate and 20% (v/v) *N*,*N*-Diisopropylethylamine (DIPEA) in DMF was applied for 10 min at room temperature. This step was repeated with a fresh solution for 40 min at room temperature. Then, the substrate was sequentially washed two times in DMF for 5 min, two times in methanol for 3 min, and in DCM for 1 min.

### Fmoc deprotection

The substrate was washed in DMF for 5 min. Then, 10 mL solution of 20% (v/v) piperidine in DMF was applied for 30 min at room temperature. The washing steps were carried out in DMF two times for 5 min each.

## Fluorescence Staining of Terminal Amino Groups

The substrate with monomer patterns was washed in Phosphate Buffered Saline with 0.05% v/v Tween® 20 (PBS-T) for 10 min and incubated in the staining solution of 5-(and-6)-Carboxytetramethylrhodamine Succinimidyl ester (TAMRA) or *DyLight* NHS 650 ester (Thermo Fisher Scientific) (1 mg/mL) in PBS-T in dilution of 1: 10 000 for 2 h in the dark. Then, the substrate was consequently washed two times in PBS-T for 3 min each, in ‘ultrapure’ water (Milli-Q) for 1 min, two times in DMF for 5 min each, two times in methanol for 3 min each and in DCM for 1 min.

## Supplementary information

Dataset 1

## Data Availability

The datasets generated during and/or analyzed during the current study are available from the corresponding author on reasonable request.

## References

[1] Fodor SPA (1991). Light-Directed, Spatially Addressable Parallel Chemical Synthesis. Science.

[2] Legutki JB (2014). Scalable high-density peptide arrays for comprehensive health monitoring. Nat. Commun..

[3] Buus S (2012). High-resolution mapping of linear antibody epitopes using ultrahigh-density peptide microarrays. Mol. Cell. Proteomics.

[4] Loeffler, F. F. *et al*. High-flexibility combinatorial peptide synthesis with laser-based transfer of monomers in solid matrix material. *Nat Commun* **7** (2016). 10.1038/ncomms11844 PMC4911634 27296868

[5] von Bojnicic-Kninski C (2016). Selective Functionalization of Microstructured Surfaces by Laser-Assisted Particle Transfer. Adv Funct Mater.

[6] Beyer M (2007). Combinatorial synthesis of peptide arrays onto a microchip. Science.

[7] Atwater Jordyn, Mattes Daniela S., Streit Bettina, von Bojničić-Kninski Clemens, Loeffler Felix F., Breitling Frank, Fuchs Harald, Hirtz Michael (2018). Combinatorial Synthesis of Macromolecular Arrays by Microchannel Cantilever Spotting (µCS). Advanced Materials.

[8] Illumina. Available online, https://www.illumina.com/science/technology/beadarray-technology.html (accessed on 15 February 2019).

[9] Nesterov-Mueller A (2014). Particle-based microarrays of oligonucleotides and oligopeptides, New and Old Technologies for Generation of Microarrays. Microarrays.

[10] Gunderson KL (2004). Decoding randomly ordered DNA arrays. Genome Res..

[11] Stadler V (2008). Combinatorial synthesis of peptide arrays with a laser printer. Angew Chem Int Edit.

[12] Michael KL (1998). Randomly ordered addressable high-density optical sensor arrays. Anal Chem.

[13] Lu Y (2001). A self-assembly approach to the fabrication of patterned, two-dimensional arrays of microlenses of organic polymers. Adv Mater.

[14] Yin YD (2001). Template-assisted self-assembly: A practical route to complex aggregates of monodispersed colloids with well-defined sizes, shapes, and structures. J Am Chem Soc.

[15] Kraus T (2007). Nanoparticle printing with single-particle resolution. Nat Nanotechnol.

[16] Brown SD (2010). Gold Nanoparticles for the Improved Anticancer Drug Delivery of the Active Component of Oxaliplatin. J Am Chem Soc.

[17] Wang Z, Wang WZ, Geng LL, Hu ZY (2015). Distinguishing of tumor cell-targeting peptide ligands through a color-encoding microarray. Lab Chip.

[18] Vafajoo, A. *et al*. Multiplexed microarrays based on optically encoded microbeads. *Biomed Microdevices* **20** (2018). 10.1007/s10544-018-0314-4 PMC6143764 30088103

[19] Popov, R. Process Development for Manufacturing Stochastic Peptide Microarrays PhD dissertation. KIT, 2018, Karlsruhe, 10.5445/IR/1000081335.

[20] Schubert Erich, Sander Jörg, Ester Martin, Kriegel Hans Peter, Xu Xiaowei (2017). DBSCAN Revisited, Revisited. ACM Transactions on Database Systems.

[21] Schirwitz, C. Purification of Peptides in High-Complexity Arrays: A New Method for the Specific Surface Exchange and Purification of Entire Peptide Libraries (Springer Theses, Springer International Publishing, 2013).

