# Supplementary material for: Stochastic deposition of amino acids into microcavities via microparticles
Source: Sci Rep. 2019 Nov 11;9:16468. doi: 10.1038/s41598-019-52994-w (PMC6848108; doi:10.1038/s41598-019-52994-w)
Supplement: Supplementary file 1 — Dataset 1 [file 41598_2019_52994_MOESM1_ESM.docx]

*Supplementary Information*

**Stochastic deposition of amino acids into microcavities via microparticles**

### Roman Popov^1^, Girish Karadka Shankara^1^, Clemens von Bojničić-Kninski^1^, Pramit Barua^1^, Daniela Mattes^1^, Frank Breitling^1^, Alexander Nesterov-Mueller^1^*

Institute of Microstructure Technology, Karlsruhe Institute of Technology (KIT), Hermann-von-Helmholtz-Platz 1, 76344 Eggenstein-Leopoldshafen, Germany; e-mail: Alexander.Nesterov-Mueller@kit.edu

|  |
| --- |
| **Figure S1.** Microbead deposition errors: (a) an unfilled microwell; (b) a microbead left on the top surface of the substrate; (c) a microbead not touching the bottom of a microwell. |

**Figure S2**: Stainless steel extraction chamber: (a) view without a lid; (b) general view; (c) view with the slide holders outside the chamber. The extraction takes place in a saturated vapor of organic solvent. The chamber consisted of two movable slide holders, each accommodating up to five substrates and two polytetrafluoroethylene tanks for the liquid-state organic solvent.

**Table S1:** Maximal content of amino acids derivatives in microbeads to achieve the mechanical stability by the deposition on microstructured surface.

| **Amino acid*** | **1-Letter notation** | **Mass fraction** | **Amino acid*** | **1-Letter notation** | **Mass fraction** |
| --- | --- | --- | --- | --- | --- |
| **Alanine** | A | 2 % | **Leucine** | L | 2 % |
| **Arginine** | R | 2 % | **Lysine** | K | 3 % |
| **Asparagine** | N | 2 % | **Methionine** | M | 3 % |
| **Aspartic acid** | D | 3 % | **Phenylalanine** | F | 3 % |
| **Cysteine** | C | 2 % | **Proline** | P | 3 % |
| **Glutamic acid** | E | 2 % | **Serine** | S | 3 % |
| **Glutamine** | Q | 2 % | **Threonine** | T | 3 % |
| **Glycine** | G | 3 % | **Tryptophan** | W | 3 % |
| **Histidine** | H | 2 % | **Tyrosine** | Y | 3 % |
| **Isoleucine** | I | 2 % | **Valine** | V | 3 % |

(*) – Fmoc-protected, OPfp-activated.

**Table S2.** Filling rates of various microstructures with microspheres (Ø 10 μm).

| **Diameter of the microwells** | **Depth of the microwells** | |
| --- | --- | --- |
|  | **9 μm** | **10 μm** |
| **11 μm** | 96.9% | 98.4% |
| **12 μm** | 90.2% | 96.3% |
| **13 μm** | 42.5% | 75.5% |

**Table S3.** Effective signal values of an experimentally derived optimal set of QDs in various fluorescence channels (in 1 000 x a.u.).

| **QDs, nm Fluorescence filter, nm/Δnm**  **520/5 549/15 580/14 615/20** | | | | |
| --- | --- | --- | --- | --- |
| **500** | **26.2 ± 10.1** | 5.9 ± 1.5 | 5.2 ± 1.1 | 12.3 ± 2.7 |
| **550** | 3.7 ± 0.9 | **40.7 ± 12.0** | 19.6 ± 5.9 | 11.4 ± 2.5 |
| **580** | 1.4 ± 1.1 | 8.0 ± 4.1 | **27.0 ± 11.8** | 18.6 ± 8.9 |
| **610** | 2.1 ± 0.4 | 6.9 ± 1.8 | 10.6 ± 2.9 | **23.5 ± 5.5** |

**Table S4.** Error rates of DBSCAN clustering.

| **QD label** | **Error rate of identifying as noise** | **Error rate of identifying as a wrong label** |
| --- | --- | --- |
| **QD 500** | 0.9 % | 0.0 % |
| **QD 550** | 6.7 % | 0.0 % |
| **QD 610** | 5.3 % | 0.0 % |
| **QD 500 + QD 550** | 3.5 % | 0.7 % |
| **QD 500 + QD 610** | 1.1 % | 0.0 % |
| **QD 550 + QD 580** | 2.0 % | 0.7 % |
| **QD 580 + QD 610** | 1.6 % | 0.0 % |
| **QD 500 + QD 550 + QD 580** | 2.3 % | 0.0 % |
| **QD 500 + QD 550 + QD 610** | 2.9 % | 0.5 % |
| **QD 500 + QD 580 + QD 610** | 5.8 % | 0.0 % |
| **QD 550 + QD 580 + QD 610** | 0.5 % | 0.0 % |

**Table S5.** Effect of extraction process duration on amino acid diffusion and coupling.

| **Duration min** | **Spot intensity,**  **1000 x a.u.** | **Spot diameter, μm** | **Spot intensity/ diameter ratio** |
| --- | --- | --- | --- |
| **0.5** | 42.5 ± 4.5 | 5.4 ± 0.4 | 7.9 |
| **1.0** | 33.2 ± 1.4 | 20.0 ± 0.9 | 1.7 |
| **2.0** | 28.5 ± 1.7 | 49.6 ± 2.9 | 0.6 |

**Table S6.** Effect of extraction scheme on amino acid diffusion and coupling.

| **Scheme, 1 min x repetitions** | **Spot intensity, 1000 x a.u.** | **Spot diameter, μm** | **Spot intensity/ diameter ratio** |
| --- | --- | --- | --- |
| **1 min x 3** | 24.3 ± 1.5 | 15.3 ± 0.9 | 1.6 |
| **1 min x 5** | 34.4 ± 2.9 | 21.9 ± 1.1 | 1.6 |
| **1 min x 7** | 31.6 ± 4.0 | 25.4 ± 1.4 | 1.2 |
| **1 min x 10** | 28.9 ± 4.4 | 28.9 ± 1.3 | 1.0 |
| **1 min x 15** | 27.8 ± 2.6 | 30.1 ± 1.9 | 0.9 |

**Table S7.** Mass concentration of amino acid derivatives in dichloromethane.

| Amino acid* | 1-Letter notation | Mass concentration, mg/ml | Amino acid* | 1-Letter notation | Mass concentration, mg/ml |
| --- | --- | --- | --- | --- | --- |
| **Alanine** | A | 5.0 | **Leucine** | L | 5.0 |
| **Arginine** | R | 5.0 | **Lysine** | K | 7.5 |
| **Asparagine** | N | 5.0 | **Methionine** | M | 7.5 |
| **Aspartic acid** | D | 7.5 | **Phenylalanine** | F | 7.5 |
| **Cysteine** | C | 5.0 | **Proline** | P | 7.5 |
| **Glutamic acid** | E | 5.0 | **Serine** | S | 7.5 |
| **Glutamine** | Q | 5.0 | **Threonine** | T | 7.5 |
| **Glycine** | G | 7.5 | **Tryptophan** | W | 7.5 |
| **Histidine** | H | 5.0 | **Tyrosine** | Y | 7.5 |
| **Isoleucine** | I | 5.0 | **Valine** | V | 7.5 |

(*) – Fmoc-protected, OPfp-activated.


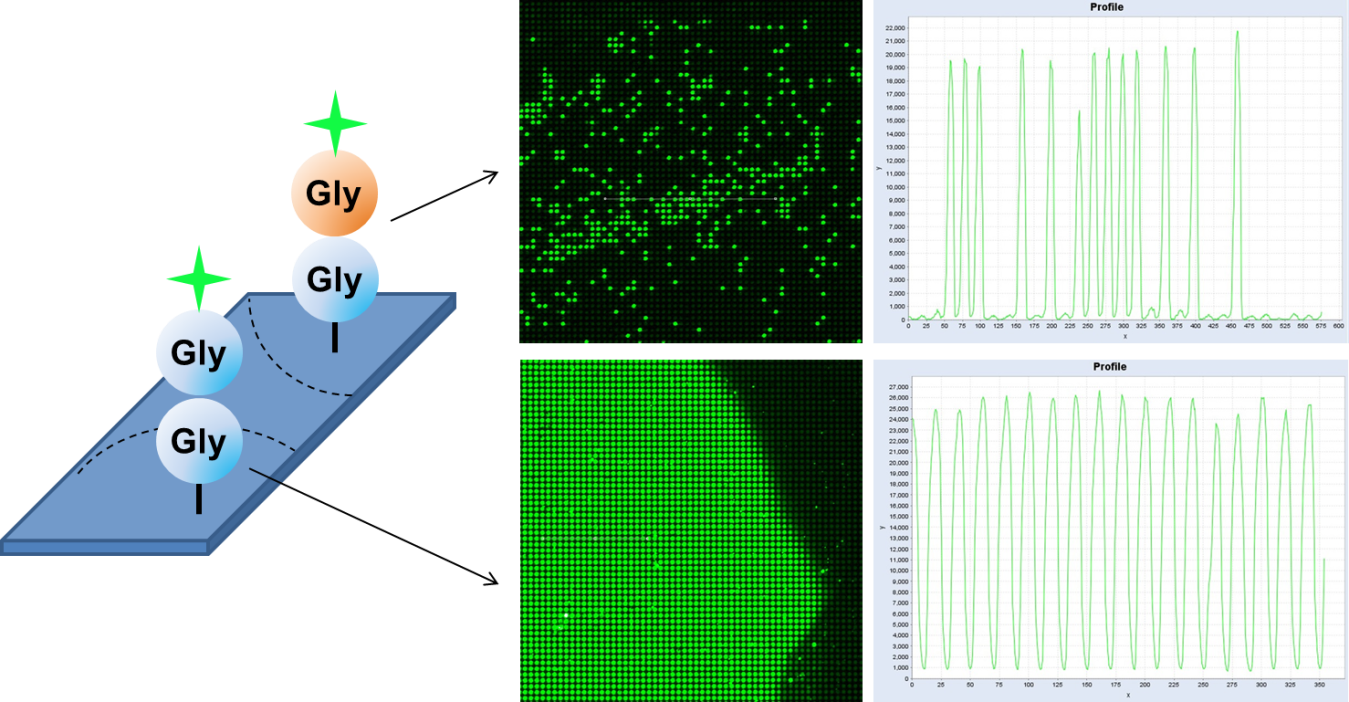


**Figure S3**: (left) The schematic representation of the dipeptide Gly-Gly synthesized from solution (blue spheres) and using particles loaded with Gly monomer (orange spheres). The first layer in both cases are synthesized from solution, while the second layer from solution and via particles. (right) Corresponding fluorescent images (green color) of the dipeptides labeled with the 5(6)-TAMRA NHS ester dye as described in section 3, Methods. **I_p_**/**I_s_** * 100% = 84%±4,7%. Here **I_p_** is the averaged intensity of the spots with the second layer synthesized via particles and **I_s_** the averaged intensity of the spots with the second layer synthesized from solution. The image was acquired at constant scanning parameters. In the experiment, following steps were taken: 1) the cavities of the glass support were functionalized with a glycine from solution. Remaining free amino groups were acetylated. The Fmoc-protecting groups were removed as described in section 2, Methods; 2) A part of the glass substrate was patterned with the Gly-particles and processed in the solvent chamber (Figure S2) as described in the main text; 3) 5 µL of 0.1M solution of Fmoc-Gly-OPfp in DMF was spotted with pipette on the other area of the glass support at room temperature; 3) The glass support was incubated in the oven as described in section 2, Methods; 4) capping and deprotection steps were applied to the glass support as described in section 2, Methods; 5) 5(6)-TAMRA NHS ester dye was coupled to the free amino groups from solution.
